# GDF9, NPHS1, and RET Mark Gastric Neuroendocrine Cells and Their Disruption in a PKA-Driven Gastric Preneoplasia Model

**DOI:** 10.3390/ijms27135642

**Published:** 2026-06-23

**Authors:** Esraa Alnahrawy, Fentahun Abate, Karl Hayden, Pawan Puri

**Affiliations:** 1 Department of Biomedical Sciences, College of Veterinary Medicine, Tuskegee University, A310 Patterson Hall, 1200 W. Montgomery Road, Tuskegee, AL 36088, USA; ealnahrawy8759@tuskegee.edu (E.A.); fabate0661@tuskegee.edu (F.A.); 2 Department of Pathobiology, College of Veterinary Medicine, Tuskegee University, 2043 Williams-Bowie Hall, 1200 W. Montgomery Road, Tuskegee, AL 36088, USA; khayden@tuskegee.edu

**Keywords:** growth differentiation factor 9 (GDF9), nephrin (NPHS1), and rearranged during transfection (RET), protein kinase A (PKA), gastric endocrine cells, gastric preneoplasia

## Abstract

The gastric endocrine population comprises functionally distinct cell types that exhibit both neuronal and endocrine characteristics; however, their molecular markers remain incompletely defined. Here, we identify growth differentiation factor 9 (GDF9), nephrin (NPHS1), and rearranged during transfection (RET) as novel markers of gastric endocrine cells. A co-immunofluorescence (IF) analysis demonstrated that GDF9, NPHS1, and RET are co-expressed with chromogranin A (CHGA), a well-known marker of gastrointestinal endocrine cells. Further Co-IF analysis revealed that GDF9-expressing cells were negative for ghrelin and somatostatin, whereas NPHS1 was co-expressed with both hormones. A subpopulation of RET-positive cells co-expressed ghrelin but not somatostatin. Notably, GDF9- and RET-positive cells co-expressed dopamine decarboxylase (DDC), consistent with enrichment in enterochromaffin-like (ECL) cells. Revisitation of our previous mRNA-sequencing data revealed reduced transcript levels of *Gdf9*, *Nphs1*, and *Ret* in CA-PKA mice, which express constitutively active protein kinase A (PKA) and develop gastric preneoplastic lesions. Co-IF and cellular quantification showed a localized reduction in the density of GDF9 and CHGA-positive endocrine cells, together with altered abundance of NPHS1- and RET-expressing cells in CA-PKA stomachs. These changes occurred in the context of extensive hyperplasia of the surrounding epithelium, indicating that the observed alterations reflect localized reduction and non-cell-autonomous effects of epithelial expansion. Notably, we observed RET misexpression outside the endocrine compartment in CA-PKA mice, suggesting that aberrant RET signaling may contribute to lesions by promoting abnormal glandular branching. Together, these findings identify GDF9, NPHS1, and RET as novel markers of gastric endocrine cells and their potential role in gastric homeostasis.

## 1. Introduction

The mouse stomach is grossly divided into two compartments: the non-glandular forestomach, which stores food and effectively regulates the availability of nutrients required for metabolism, and the glandular stomach, which is responsible for digestion by secreting acid and enzymes [1,2,3,4,5,6,7,8]. In addition, the glandular stomach acts as an endocrine organ, releasing various hormones. Both compartments are separated by a limiting ridge (margo plicatus) [9,10]. In contrast to the murine stomach, the human stomach and the stomachs of other monogastric species, such as dogs, are entirely glandular, as the glandular mucosa also lines the proximal cardia region [11].

The mouse glandular stomach is composed of two anatomically and functionally distinct regions: the corpus and the antrum (also referred as the pyloric region) [1,12]. The corpus contains specialized epithelial cells, including pit cells and mucous neck cells (which secrete mucus), parietal cells (which secrete hydrochloric acid), and chief cells (which produce digestive enzymes, including gastric intrinsic factor) [1,12]. The antrum or the pyloric region includes the pyloric antrum (antrum pyloricum), and the pyloric canal (canalis pyloricus) [11,13,14]. The antrum pyloricum is the region adjacent to the gastric corpus, while the canalis pyloricus extends toward the pyloric sphincter [15]. The antrum is mainly composed of mucus-secreting epithelial cells.

The glandular stomach also consists of functionally diverse endocrine cell types, including histamine-secreting enterochromaffin-like (ECL) cells, somatostatin-secreting D cells, ghrelin-secreting X/A cells, serotonin-secreting mast cells, and gastrin-secreting G cells, which contribute to the regulation of gastric motility, acid production, and overall epithelial homeostasis [1]. These endocrine cells share phenotypic features of both neuronal and endocrine lineages, enabling neuronal, endocrine, and paracrine modes of communication [16,17]. Structurally, the gastric endocrine population has hormone-containing large dense core vesicles (LDCVs) and is also known to contain synaptic-like micro vesicles (SLMV) [18,19]. However, the molecular markers underlying their functional diversity remain incompletely defined.

Bone morphogenetic protein (BMP) signaling is known to regulate the number and differentiation of neuroendocrine cells of the stomach and intestine [20]. In the intestine, BMP signaling regulates the endocrine secretory features of neuroendocrine cells along the crypt–villus axis [21]. BMP signaling is one of the pathways of the transforming growth factor beta (TGFβ) family that encompasses a broad group of ligands, including TGFβ isoforms, activins, nodal, BMPs, and growth differentiation factors (GDFs) [22]. While the roles of TGFβs, BMPs, activins, and nodal have been relatively well studied in the stomach, the expression and function of GDF ligands remain largely unexplored [23,24,25,26,27,28,29,30]. GDF15, a distant member of the TGFβ superfamily, has been implicated in gastric cancer, food intake and obesity; however, the role of other GDFs in gastric biology remains unknown [31,32,33,34]. GDF9 is highly enriched in the ovary, where it plays a key role in folliculogenesis alongside BMP15 [35]. GDF9 is known to signal through ALK5 and BMPRII to activate SMAD2/3 in the ovary [36]. A few studies have reported GDF9 expression in other organs, such as the kidney and liver, but its expression and function in the stomach have not been previously characterized [37,38].

In earlier work, we generated and analyzed a conditional mouse model expressing constitutively active protein kinase A (PKA) (CA-PKA) in the gastric mesenchyme [39]. These mice develop gastric preneoplastic lesions characterized by marked epithelial hyperplasia, metaplasia, cyst formation, and inflammation. Bulk RNA sequencing revealed dysregulation of BMP and SMAD signaling pathways in the CA-PKA stomach. Interestingly, revisitation of our RNA-sequencing data revealed significant downregulation of *Gdf9*, prompting us to investigate its expression and localization in control and CA-PKA mutant stomachs.

Recent single-cell RNA-sequencing studies have identified genes enriched in enteroendocrine cells of the human gastrointestinal tract including stomach [40,41]. Surprisingly, *Nphs1*, a gene highly expressed in renal podocytes and essential for the integrity of the glomerular filtration barrier was also found to be enriched in gastric endocrine cells [40]. Mutations in *Nphs1*, which cause congenital nephrotic syndrome, have been associated with gastric lesions, including glandular duplication cysts [42]. These findings prompted us to investigate NPHS1 expression and localization in the stomachs of control and CA-PKA mice.

Rearranged during transfection (RET) is a proto-oncogene that plays an indispensable role in the differentiation of enteric neurons and in the branching morphogenesis of the ureteric bud in the developing kidney [43]. Recently, RET has been shown to be expressed in enterochromaffin (EC) cells in the intestine and regulates maturation, branching at the cellular level and intestinal motility at the physiological level [44,45,46]. Although the expression and function of RET in the intestine is now established, its expression in the stomach has not been explored.

In the CA-PKA model, we observed downregulation of several endocrine-enriched markers, including *Chga*, *Hdc* (histidine decarboxylase) and *Sst* (somatostatin), at the transcript level [39]. However, it remained unclear whether these changes reflected transcriptional suppression, loss of gastric endocrine cells, or it represents relative decrease due to massive epithelial hyperplasia. The main aim of this study is to characterize the expression and localization of novel markers (GDF9, NPHS1, and RET) in gastric neuroendocrine cell populations and to evaluate changes in these markers under preneoplastic conditions in the CA-PKA mouse model.

## 2. Results

**Figure 1:**
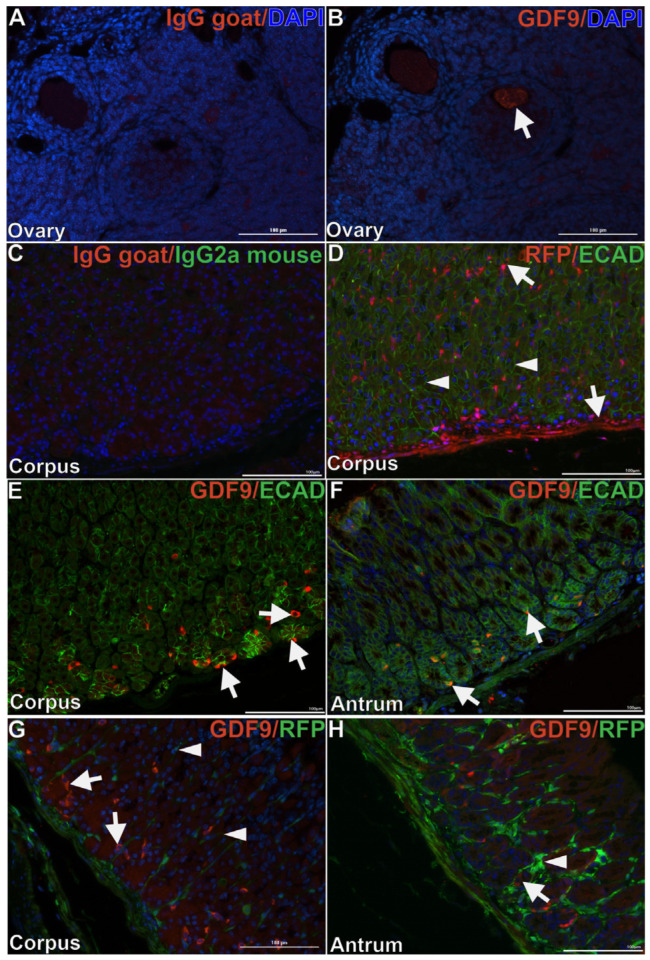
Representative Co-IF images showing GDF9 expression in the epithelial compartment of the stomach. (**A**,**B**) (**A**) Negative control (ovary) stained with IgG goat antibody (isotype control) shows minimal background fluorescence. (**B**) Ovary sections stained with GDF9 antibody show GDF9-positive germ cells (red; arrow) with DAPI (blue). (**C**,**D**) (**C**) Merged image of gastric corpus sections stained for goat IgG (red) and mouse IgG2a (green), and DAPI (blue) shows minimal to no non-specific signal. (**D**) Co-IF staining for tdTomato (red) and the epithelial marker E-cadherin (ECAD, green) in the gastric corpus. Arrows indicate tdTomato-positive mesenchymal cells and arrowheads indicate ECAD-positive epithelial cells. (**E**,**F**) Co-IF staining for GDF9 (red) and the epithelial marker E-cadherin (ECAD, green) in the gastric corpus (**E**) and antrum (**F**). The arrows indicate GDF9-positive epithelial cells. (**G**,**H**) Gastric corpus (**G**) and antrum (**H**) stained with antisera against GDF9 (red) and tdTomato (green), marking the mesenchymal reporter lineage. The arrows indicate GDF9-positive cells within the glandular region, whereas arrowheads denote RFP-positive mesenchymal cells. DAPI (blue). Scale bar: 100 µm.

To begin, we tested the specificity of the GDF9 antibody through IF analysis on mouse ovary sections. The IF staining with GDF9 antisera revealed a specific signal localized to the female germ cells; in contrast, minimal background fluorescence was observed in the section stained with goat IgG control, confirming the specificity of the GDF9 antibody (Figure 1A,B).

To study gastric GDF9 expression, we used stomachs isolated from *Six2*-Cre^+/−^;tdTomato^+/−^ reporter mice [39]. In this model, *Six2* promoter-driven Cre recombination permanently labels *Six2*-expressing progenitor-derived cells with tdTomato, which are restricted to the mesenchymal compartment of the stomach. We performed Co-IF staining using antibodies against red fluorescent protein (RFP), which detects tdTomato, and the epithelial cell marker E-cadherin (ECAD). As controls, stomach sections from *Six2*-Cre^+/−^;tdTomato^+/−^ mice were probed with mouse IgG2a and goat IgG isotype control antibodies. With isotype controls, minimum to no non-specific fluorescence signal was detected, confirming staining specificity (Figure 1C). RFP staining was restricted to the mesenchymal compartment, whereas ECAD staining was specifically localized to the epithelial compartment, confirming their distinct compartmental distribution within the adult stomach from *Six2*-Cre^+/−^;tdTomato^+/−^ mice (Figure 1D). Further, Co-IF analysis with GDF9 and ECAD antibodies in mouse stomach revealed that GDF9-positive cells were localized within the epithelial glands of the stomach (Figure 1E,F). To determine whether mesenchymal cells express GDF9, we performed co-staining with antibodies against GDF9 and tdTomato. GDF9 expression was undetectable in tdTomato-positive cells, which represent a broader *Six2*-derived mesenchymal cells (Figure 1G,H). These results confirmed the glandular-specific expression pattern of GDF9 in the stomach.

**Figure 2:**
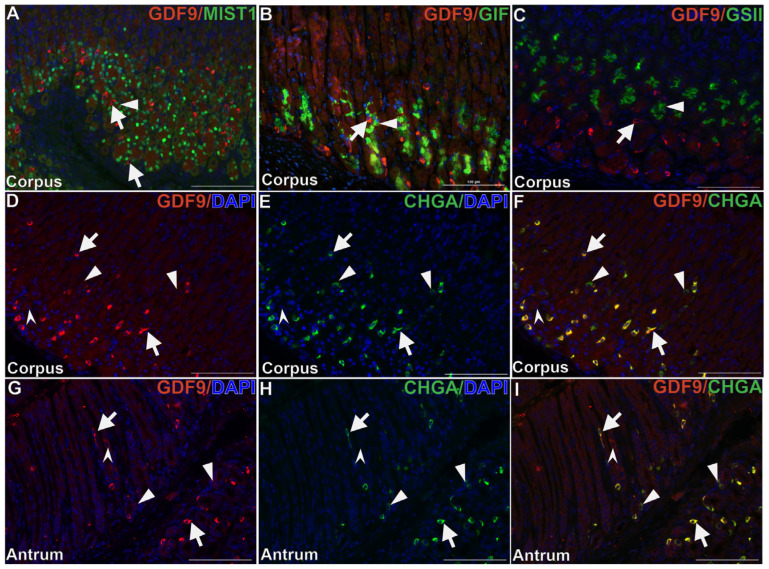
GDF9 is co-expressed in CHGA-positive gastric endocrine cells in the mouse corpus and antrum. (**A**–**C**) Co-IF staining in the gastric corpus for GDF9 (red) with chief cell markers, MIST1 (green) (**A**) and GIF (**B**), and mucous neck cell marker, GSII-lectin (green) (**C**). The arrows indicate GDF9-positive cells, and the arrowheads indicate cells positive for the indicated lineage markers. No overlap is observed between GDF9 and markers of chief cells and mucous neck cells. (**D**–**F**) Gastric corpus sections stained for GDF9 (red) and pan-endocrine marker (CHGA, green) and the merged image showing co-localization of GDF9 and CHGA (**F**). (**G**–**I**) Gastric antrum sections stained for GDF9 (red) and CHGA (green) and the merged image showing co-localization of GDF9 and CHGA (**I**). The arrows indicate GDF9-positive/CHGA-positive endocrine cells, whereas the arrowhead indicates GDF9-negative/CHGA-positive cells. The concave arrowhead indicates GDF9-positive/CHGA-negative cells. The nuclei are counterstained with DAPI (blue). Scale bars, 100 μm.

We noticed that GDF9-positive cells were predominantly localized near the glandular base, where chief cells, mucus neck cells, parietal cells, and endocrine cells are typically found. To investigate whether chief cells express GDF9, we co-stained stomach sections with transcription factor MIST1 and gastric intrinsic factor (GIF), which are well-established markers of murine chief cells [12]. Co-IF analysis demonstrated that MIST1- and GIF-positive cells did not express GDF9 (Figure 2A,B). To assess whether mucus neck cells express GDF9, we co-stained with GSII lectin, a marker for these cells. This analysis showed that GSII-positive mucus neck cells were also GDF9-negative (Figure 2C). Finally, we evaluated the expression of GDF9 in gastric endocrine cells, which are marked by chromogranin A (CHGA). Co-IF analysis showed that, in both corpus and antrum, nearly all GDF9-positive cells were CHGA-positive. (Figure 2D–I). A very small subset of CHGA-negative cells showed weak GDF9 expression, and a few CHGA-positive cells were GDF9-negative (Figure 2D–I). Together, these findings indicate that GDF9 expression in the stomach is restricted to the epithelial compartment, and more specifically, GDF9 marks endocrine cells within the gastric glands.

**Figure 3:**
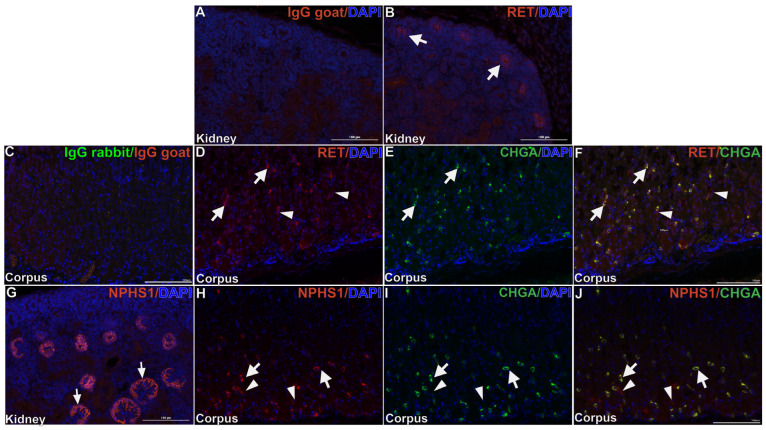
RET and NPHS1 are co-expressed in CHGA-positive gastric endocrine cells in the mouse corpus. (**A**,**B**) (**A**) Kidney sections stained with IgG goat (isotype control) shows minimal background fluorescence signal. (**B**) Kidney sections stained with RET antibody show fluorescent signal for RET (red) in the ureteric bud (arrows) with DAPI (blue). (**C**) Merged image of gastric corpus sections stained for goat IgG (red) and rabbit IgG (green) and DAPI (blue) shows minimal to no background fluorescence signal. (**D**–**F**) Gastric corpus sections stained for RET (red) and CHGA (green) and the merged image showing co-localization of RET and CHGA. The arrows indicate RET-positive/CHGA-positive endocrine cells, whereas the arrowheads indicate positive cells with variable RET expression. (**G**) Kidney sections stained with NPHS1 antibody show fluorescent signal for NPHS1 (red) in podocytes of renal glomeruli (arrows) with DAPI (blue). (**H**–**J**) Gastric corpus sections stained for NPHS1 (red) and CHGA (green) and the merged image showing co-localization of NPHS1 and CHGA. The arrows indicate NPHS1-positive/CHGA-positive endocrine cells, whereas the arrowheads indicate cells positive for only one marker.

We tested the specificity of the RET antibody in the kidney. RET fluorescence was found to be enriched in the uretic bud cells of the developing kidney, whereas the control kidney sections stained with goat IgG exhibited minimal background fluorescence, confirming antibody specificity (Figure 3A,B). Next, we performed a Co-IF experiment with RET and CHGA antibodies to determine whether RET is expressed in gastric endocrine cells. For controls, stomach sections were stained with rabbit IgG and goat IgG isotype antibodies, and minimal to no background fluorescence signal was detected (Figure 3C). We found that RET is expressed in CHGA-positive endocrine cells in the mouse stomach (Figure 3D–F). We also observed a very small subset of RET-positive, CHGA-negative cells (Figure 3D–F).

Next, we tested the specificity of the NPHS1 antibody in the kidney. IF staining of kidney sections from neonatal mice was performed using an NPHS1 antibody. NPHS1 staining was specifically localized to podocytes within developing glomeruli, confirming antibody specificity (Figure 3G). Next, we used NPHS1 in combination with CHGA to determine whether gastric endocrine cells in the mouse stomach express NPHS1. Co-IF analysis with NPHS1 and CHGA antibodies revealed that NPHS1 is indeed expressed in CHGA-positive cells (Figure 3H–J). Additionally, we observed very few NPHS1-positive, CHGA-negative cells, suggesting that NPHS1 may also be expressed in other cell types within the gastric epithelium (Figure 3H–J). Together, these findings demonstrate that NPHS1, RET, and GDF9 mark the gastric endocrine cell population, highlighting a previously unrecognized molecular signature of these neuroendocrine cells in the stomach.

**Figure 4:**
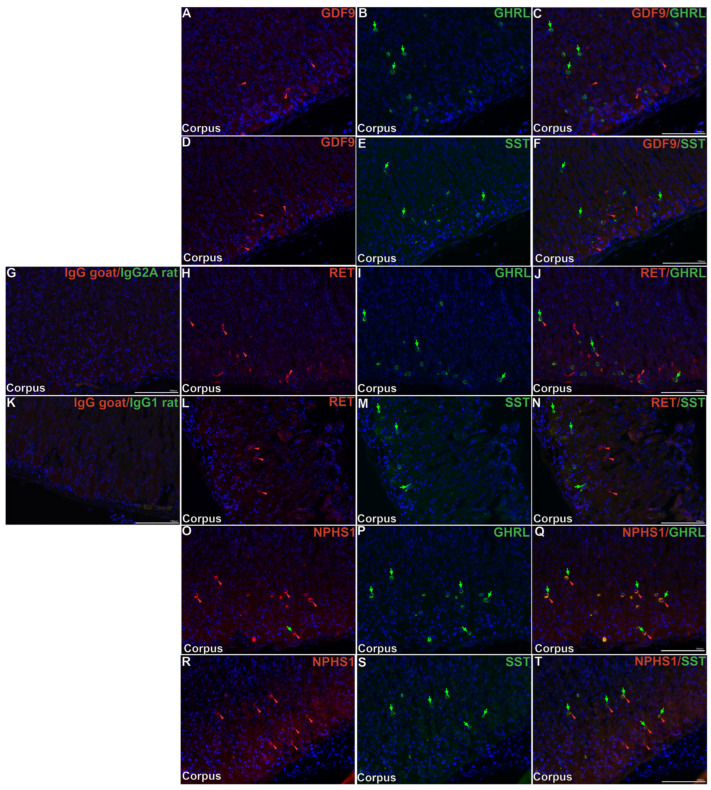
GDF9, RET, and NPHS1 expression in gastric endocrine subpopulations in the mouse corpus. Representative IF images of mouse gastric corpus sections showing co-expression patterns of GDF9, RET, and NPHS1 with the endocrine hormones ghrelin (GHRL) and somatostatin (SST). (**A**–**C**) GDF9 (red), GHRL (green), and merged image. (**D**–**F**) GDF9 (red), SST (green), and merged image. (**G**) Merged image of gastric corpus sections stained with goat IgG (red), rat IgG2a (green), and DAPI (blue). (**H**–**J**) RET (red), GHRL (green), and merged image. (**K**) Merged image of gastric corpus sections stained for goat IgG (red), rat IgG1 (green), and DAPI (blue). (**L**–**N**) RET (red), SST (green), and merged image. (**O**–**Q**) NPHS1 (red), GHRL (green), and merged image. (**R**–**T**) NPHS1 (red), SST (green), and merged image. The green arrows indicate ghrelin or somatostatin expression, the red arrowheads indicate GDF9, RET, or NPHS1 expression, and both the green and the red arrowheads indicate double-positive cells in the merged images. The nuclei are counterstained with DAPI (blue). Scale bars, 100 μm.

To define the gastric endocrine subpopulation expressing GDF9, we first performed Co-IF staining using antibodies against GDF9, together with either ghrelin or somatostatin. These co-IF experiments demonstrated that almost all of GDF9-positive cells were negative for both ghrelin (Figure 4A–C) and somatostatin (Figure 4D–F). We also performed isotype control studies for ghrelin co-localization experiments using rat IgG2a and goat IgG controls (Figure 4G) and somatostatin (Sst) co-localization studies using rat IgG1 and goat IgG controls (Figure 4K). In both corresponding isotype control sections, minimum to no non-specific fluorescence staining was detected. We also performed Co-IF studies to determine whether RET-positive gastric endocrine cells express ghrelin or somatostatin. We found that a subpopulation of RET- and ghrelin-double-positive cells exists, and these cells are weakly positive for RET (Figure 4H–J), whereas no co-expression of RET with somatostatin was observed (Figure 4L–N). NPHS1 is known to regulate insulin secretion in the pancreas [47]. Consistent with this role in hormone secretion, our Co-IF experiments showed that NPHS1 is widely co-expressed with ghrelin (Figure 4O–Q) and is also co-expressed with somatostatin, albeit at a lower frequency (Figure 4R–T).

**Figure 5:**
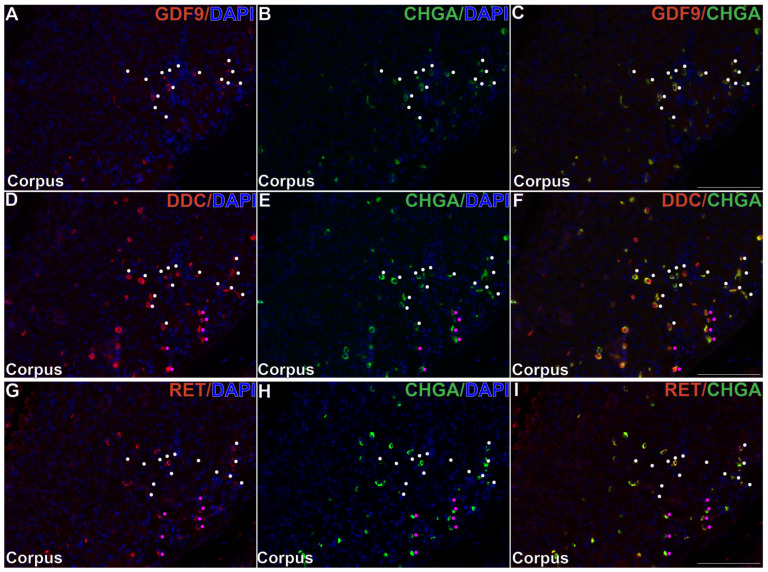
Representative Co-IF staining of serial sections from the mouse gastric corpus showing GDF9/CHGA expression and its spatial association with DDC/CHGA- and RET/CHGA-expressing cells. (**A**–**C**) Co-IF for GDF9 (red) and CHGA (green) with DAPI (blue). The white dots indicate GDF9 and CHGA expression and regions of overlap with panels underneath. (**D**–**F**) Serial sections stained with DDC (red), with CHGA (green) and DAPI (blue). The white and magenta dots denote DDC and CHGA expression in cells with regions overlapping those shown in the panels above and below (**G**–**I**). Co-IF for RET (red) and CHGA (green) with DAPI in adjacent sections. The white and magenta dots denote RET and CHGA expression in cells with regions overlapping those shown in the panels above. Scale bars, 100 μm.

Next, we performed Co-IF staining for GDF9/CHGA, DDC/CHGA, and RET/CHGA on serial sections of gastric corpus tissue to determine whether GDF9- and RET-expressing gastric endocrine cells are associated with the dopamine decarboxylase (DDC), a key enzyme involved in the biosynthesis of neurotransmitters and a marker of ECL cells. Because antisera against GDF9, DDC, and RET were all generated in goat, co-expression studies on a single tissue section were not feasible; therefore, serial sections were analyzed. Serial section analysis revealed that GDF9- and RET-positive endocrine cells aligned with DDC/CHGA double-positive cells within the same glandular regions, indicating that these markers represent overlapping subsets of the gastric ECL population (Figure 5A–I). The spatial correspondence of GDF9-, DDC-, and RET-positive cells across serial sections suggests the existence of an endocrine subpopulation with shared expression of these markers.

**Figure 6:**
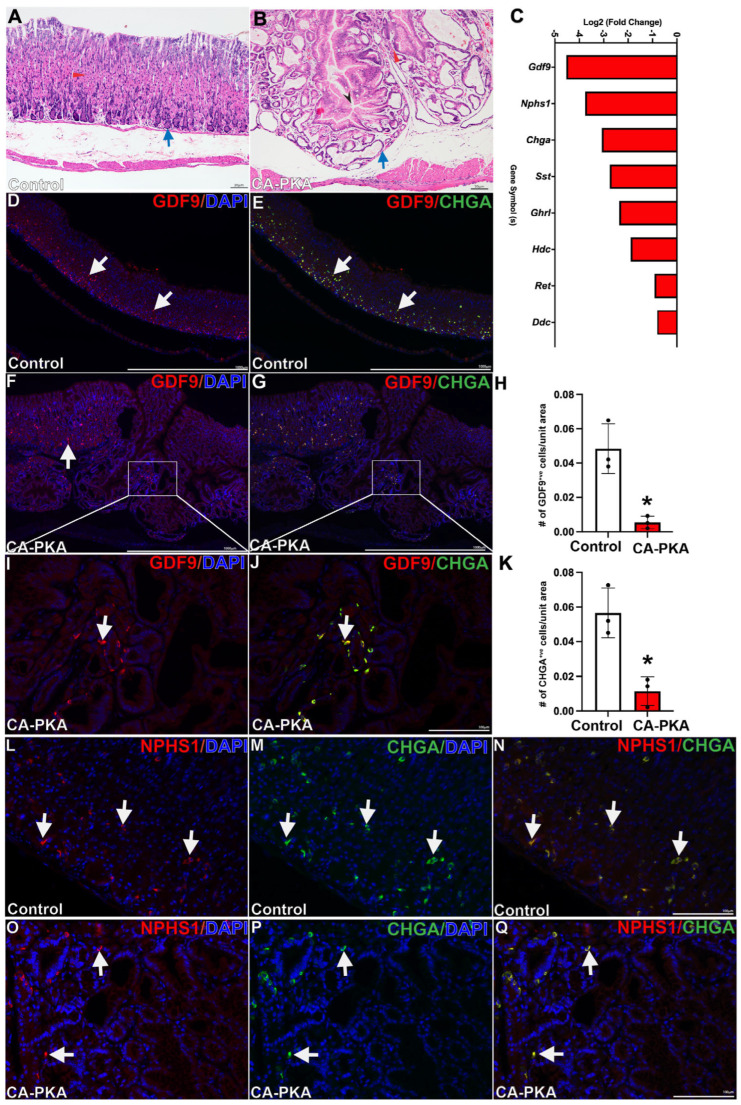
CA-PKA mice show alterations in gastric endocrine cell populations. (**A**,**B**) (**A**) Hematoxylin and eosin stain sections from control and CA-PKA mice. The arrow indicates chief cells at the base of the gland, and the arrowhead indicates parietal cells. (**B**) In the mutant stomach, the base of the gland is cystic, parietal cells are reduced, and the concave arrowhead indicates branching glands. (**C**) Revisitation of the previously published bulk mRNA sequencing (#GSE196236) identified alterations in the transcript levels enriched in gastric endothelial cells in the control and CA-PKA mutant corpus. All data with red bars show significant gene changes, with a *p* value of 0.05 or less. (**D**,**E**) Co-IF staining of control gastric corpus sections showing GDF9 (red) expression that partially overlaps (arrows) with the endocrine cell marker CHGA (green). DAPI marks nuclei (blue). (**F**,**G**) GDF9 and CHGA Co-IF in CA-PKA stomachs indicating reduction and mis-localization of GDF9- and CHGA-positive cells. Enlarged insets (**I**,**J**) highlight the reduced number of GDF9- and CHGA-positive cells in CA-PKA mice (arrows). (**H**,**K**) Quantification of GDF9-positive (**H**) and CHGA-positive (**K**) cells per unit area show significant decrease in CA-PKA stomachs compared with the controls. Data represent mean ± SEM; * *p* < 0.05. (**J**–**O**) Co-IF staining of the control (**L**–**N**) and CA-PKA (**O**–**Q**) stomachs with NPHS1 (red) and CHGA (green) shows loss of NPHS1 and CHGA-positive endocrine cells. Arrows indicate NPHS1- and CHGA-positive cells in the respective individual panels and the merged image.

Next, we were interested in studying the expression of these markers in CA-PKA mutants. Hematoxylin and eosin staining of stomach sections from CA-PKA mutant mice showed marked gastric gland hyperplasia and cystic glandular dilation compared with the normal glandular architecture observed in the control stomachs (Figure 6A,B). We revisited our previously published RNA-seq dataset (#GSE196236) to assess whether other endocrine-enriched transcripts, including *Gdf9*, *Nphs1*, and *Ret*, were altered in the corpus region of CA-PKA mutant mice compared to controls. Revisiting the differentially expressed gene list previously published from bulk mRNA seq of control and CA-PKA mice revealed significant downregulation of multiple endocrine-enriched genes in the mutant corpus, including *Gdf9*, *Nphs1*, *Ret*, *Chga*, *Chgb*, *Ghrl*, *Sst*, *Hdc*, and *Ddc* (Figure 6C). *Chga*, *Hdc,* and *Sst* were highlighted in our previously published study [39]. To validate these transcriptomic findings at the protein level, we performed Co-IF analyses for GDF9 and CHGA. In the control stomach, GDF9- and CHGA-positive endocrine cells were distributed predominantly within the lower half of the gastric glands (Figure 6D,E). In contrast, the corpus region of CA-PKA mutant mice showed an uneven distribution and localized loss of GDF9- and CHGA-positive cells, with a marked reduction in areas displaying pronounced cystic and hyperplasic lesions, whereas regions with comparatively preserved glandular organization retained the GDF9-positive cells (Figure 6F,G,I,J). The quantification of endocrine cells normalized to tissue surface area revealed a significant reduction in the density of GDF9-positive cells in CA-PKA mutants (Figure 6H). Consistent with this finding, the density of CHGA-positive endocrine cells was also reduced in mutant stomachs, although to a lesser extent than that of GDF9-positive cells, indicating differential sensitivity among endocrine subpopulations to modified mesenchymal microenvironments (Figure 6K). In addition to changes in cell density, endocrine cells in CA-PKA mutants frequently exhibited altered spatial localization, with many cells displaced from their normal position in the basal half of the gastric glands toward the upper third of the gland (Figure 6F). We also performed Co-IF analyses to examine the status of NPHS1-positive cells in stomachs isolated from CA-PKA mice. A similar pattern was observed for NPHS1, with localized loss of NPHS1-positive endocrine cells in the affected gastric glands (Figure 6L–Q).

**Figure 7:**
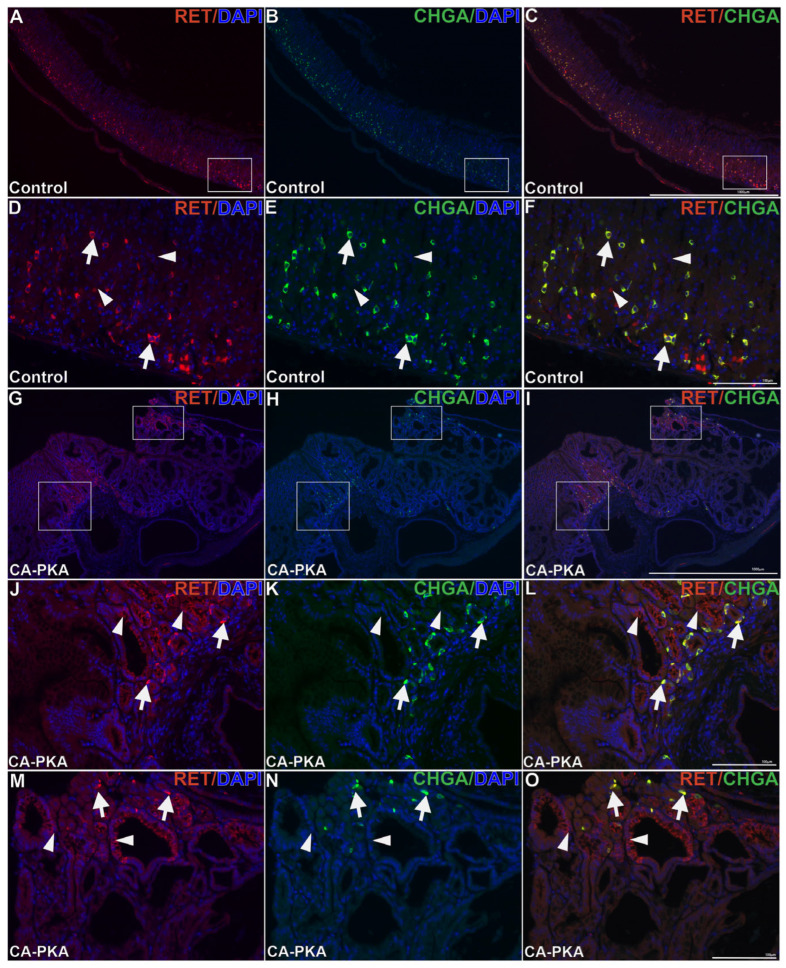
Alteration in RET-positive CHGA-negative population in CA-PKA stomach. (**A**–**F**) Co-IF staining of control stomachs with RET (red) and CHGA (green) at 4X magnification (**A**–**C**) and corresponding 20X inset images (**D**–**F**). RET and CHGA co-expression was observed in gastric endocrine cells. The arrows indicate CHGA-positive/RET-positive cells. The arrowheads indicate a population of CHGA-negative cells with relatively weak RET expression. (**G**–**O**) Co-IF staining of CA-PKA stomachs with RET (red) and CHGA (green) at 4X magnification (**G**–**I**) and corresponding two 20X inset images (**J**–**O**). The arrows indicate CHGA-positive/RET-positive cells. Ret immunoreactivity was observed in CHGA-negative cells (arrowheads), and this population appeared more prominent in CA-PKA stomachs.

Co-IF analysis was performed to examine the distribution of RET-positive cells in stomachs from control and CA-PKA mice. In the control stomachs, RET expression showed variable intensity, with strong RET fluorescence predominantly observed in CHGA-positive endocrine cells (Figure 7A–F). A small population of CHGA-negative/RET-positive cells was also detected, although RET fluorescence intensity in these cells was relatively low (Figure 7A–F). In the CA-PKA mutant stomachs, CHGA-positive/RET-expressing endocrine cells appeared reduced within cystic and hyperplastic epithelial regions (Figure 7G–O). In addition, RET immunoreactivity was observed in a population of CHGA-negative cells, and this population appeared more prominent in CA-PKA stomachs (Figure 7G–O). Collectively, these results show that gastric mesenchymal PKA activation is accompanied by coordinated changes in the density, regional loss and spatial disorganization of multiple gastric endocrine cell populations, occurring in parallel with extensive epithelial remodeling.

## 3. Discussion

Our study identifies GDF9, NPHS1, and RET as novel markers of murine gastric endocrine cells and further defines their expression across distinct gastric endocrine subpopulations. We also demonstrate that expression of these markers, along with the associated endocrine cell populations, is altered in the stomachs of CA-PKA mice that develop preneoplastic lesions.

Co-expression of the majority of GDF9-, NPHS1-, and RET-positive cells with CHGA, a well-established marker of gastric endocrine cells, shows that these proteins localize predominantly to the gastric endocrine compartment. Lack of co-expression of GDF9 with ghrelin or somatostatin shows the distinct identity of GDF9-expressing cells from ghrelin-secreting X/A cells and somatostatin-secreting D cells. Histidine decarboxylase (HDC) is a well-established marker of enterochromaffin-like (ECL) cells, and HDC-positive ECL cells have been reported to co-express both ghrelin and somatostatin [48]. However, in our hands, a subpopulation of cells with relatively weak RET expression expressed ghrelin; however, the RET-positive population was negative for somatostatin. GDF9- and RET-positive cells were found to align spatially with DDC-expressing cells in serial sections, suggesting co-expression. DDC is more broadly expressed and catalyzes the decarboxylation of monoamines to generate neurotransmitters such as dopamine and serotonin [49]. Although single-cell mRNA-sequencing studies have shown enrichment of *Ddc* expression in ECL cells within the mouse gastric corpus, in our analyses, DDC immunoreactivity was detected in most CHGA-positive cells, albeit with variable intensity [41]. Together, these findings suggest that GDF9-positive cells likely represent a distinct subpopulation within the broader ECL cell compartment. Another possibility is that subsets of GDF9-positive or RET-positive cells represent gastric mucosal mast cells, which are bone marrow-derived, lack classical endocrine markers, and are known to reside within the gastric mucosa, where they express monoamine-related enzymes [48].

*Gdf9* transcript levels were found to be markedly downregulated in the stomach of CA-PKA mice, a finding revealed by revisitation of our previously published bulk RNA-sequencing data [39]. We also showed that CA-PKA stomachs exhibit reduced BMP signaling and decreased SMAD1/5/8 activity [39]. GDF9 is a TGF-β family ligand that activates SMAD2/3 signaling via BMPRII and TGFBR1 to regulate folliculogenesis in the ovary [36]. A recent study reported luteinizing hormone (LH) expression in gastric enterochromaffin-like (ECL) cells, highlighting the stomach as an extraovarian site for proteins classically associated with reproductive endocrine tissues [41]. Notably, deletion of *Smad3* in the stomach induces gastric carcinogenesis and spasmolytic polypeptide-expressing metaplasia (SPEM), a key feature of CA-PKA preneoplastic lesions [50,51]. Future studies will be required to determine whether GDF9 activates SMAD signaling in the stomach, and whether localized loss of GDF9 expression in CA-PKA mice contributes to the development of SPEM lesion. In addition, GDF15, another member of the GDF family, regulates food intake and energy balance through GFRAL-RET signaling [33,34]. Notably, our finding that GDF9 and RET are co-expressed within the gastric endocrine population raises the possibility that GDF9-RET-dependent signaling may operate in the stomach in a manner analogous to GDF15 signaling.

Recent single-cell RNA-sequencing of the human stomach identified *Nps1* as enriched in gastric enterochromaffin cells [40]. We show that NPHS1 protein is expressed in murine gastric endocrine cells and co-localizes with both ghrelin- and somatostatin-positive populations. *Nphs1* is best known for its essential role in podocytes, where loss-of-function mutations cause congenital nephrotic syndrome, but it also regulates insulin secretion in pancreatic β cells, indicating a broader role in hormone secretion [47,52]. Consistent with this function, NPHS1 expression in gastric endocrine cells suggests involvement in gastric hormone release. Notably, NPHS1 has been reported to be expressed in the stomach, and *Nphs1* mutations are associated with gastric duplication cysts [42]. How the localized loss of NPHS1-positive endocrine cell population observed in CA-PKA mutant stomachs is linked to cystic lesion formation will require further investigation.

RET is a well-established regulator of branching morphogenesis in the kidney [43,53]. In the intestine, RET expression is largely restricted to enterochromaffin (EC) cells, where it has been implicated in epithelial branching and maturation [44,45,46]. In CA-PKA mutant stomachs, RET-positive/CHGA-positive endocrine cells showed localized reduction; however, we also observed that RET-positive/CHGA-negative cells appeared relatively increased within cystic and hyperplastic glands exhibiting excessive branching and disrupted architecture. In control stomachs, RET-positive/CHGA-negative cells were relatively sparse, and Ret fluorescence intensity within these cells was comparatively weak, limiting rigorous quantitative assessment. RET expression has been reported to be upregulated in gastric adenocarcinoma however, the specific gastric cell population has not been defined [54]. However, the analysis of the Human Protein Atlas single-cell database indicated that although *Ret* expression in the stomach is enriched in endocrine populations, *Ret* transcripts are also detected in additional gastric epithelial and mesenchymal cell types, including mucus cells, parietal cells, pit cells and pericytes [55,56].

Importantly, interpretation of the co-IF findings, together with the bulk RNA-sequencing data, may provide additional context, as RNA-seq demonstrated substantially greater downregulation of *Gdf9* transcripts compared with *Ret* transcripts, despite the observation that a large proportion of GDF9- and RET-positive cells are CHGA-positive. Therefore, multiple possibilities could explain the differential regulation of these markers, including relative expansion of a RET-positive/CHGA-negative population, increased RET- expression in previously RET-low cells, or differential transcript sensitivity to the altered microenvironment. While bulk RNA-seq cannot definitively resolve cell-specific expression changes, the combined Co-IF and transcriptomic findings support the possibility that RET-positive/CHGA-negative non-endocrine populations may be relatively expanded in the mutant stomach environment. Given the conserved role of RET in branching morphogenesis, these findings further raise the possibility that altered RET expression may contribute to abnormal glandular remodeling and gastric hyperplasia in CA-PKA mice.

Other key observations we made in this study were concomitant decreases in transcripts encoding *Gdf9*, *Nphs1*, *Ret*, *Ghrl*, *Sst*, *Chga*, *Chgb*, *Hdc*, and *Ddc*, along with mislocalized and depleted gastric endocrine cells in CA-PKA stomach. Collectively, these findings suggest that the niche supporting gastric endocrine cells is likely disrupted, as a consequence of the extensive expansion and remodeling of both the mesenchyme and epithelium. BMP signaling has been shown to regulate endocrine cell positioning along the crypt–villus axis in the intestine [21]. Our previous work demonstrated that constitutive activation of PKA in the mesenchyme perturbs mesenchymal–epithelial crosstalk, with downregulation of multiple components of the BMP signaling pathway representing a major molecular alteration [39]. These changes are therefore likely to contribute to the loss and mislocalization of gastric endocrine cells and, in parallel, to promote ectopic RET-dependent branching. Together, our findings link altered endocrine homeostasis to hyperplastic mesenchymal and glandular remodeling in the CA-PKA stomach.

One of the limitations of the present study is that the Co-IF analyses were primarily qualitative and were not designed for comprehensive quantitative assessment of all cellular populations. Interpretation of co-localization data was additionally complicated by the relatively low abundance of certain cell populations and variable expression levels of these markers in the stomach. In some cases, weaker fluorescence intensity made it difficult to confidently distinguish low-expressing cells from background staining, thereby limiting rigorous quantification and direct comparison between the cell types in control and mutant stomachs. Importantly, increasing evidence obtained from advanced imaging and transcriptomics technologies suggests that enteroendocrine cells are not static single-hormone-expressing populations but instead exhibit dynamic and heterogeneous hormone and marker expression profiles, including overlapping and variable high- and low-expressing cellular and subcellular states [57,58,59,60,61,62,63]. These biological features further complicate interpretation and quantification using conventional co-IF approaches alone. Furthermore, simultaneous multi-marker co-expression analyses were technically restricted because several primary antibodies (GDF9, RET, NPHS1 and DDC,) were generated in the same host species (goat), limiting the use of standard multiplex immunofluorescence approaches. Future studies using higher-resolution approaches such as spatial transcriptomics, single-cell RNA sequencing, multiplex imaging platforms, proteomics, and transgenic reporter models will be important to further define the identity and functional significance of these gastric cell populations.

## 4. Materials and Methods

### 4.1. Generation of Conditional Mutant Mice

The generation of the CA-PKA and *Six2*-Cre mouse lines and genotyping strategies were previously described [39]. Mice harboring a constitutively active mutation in the *Prkaca* gene encoding the catalytic subunit α of protein kinase A (PKAcα) (hereafter referred to as PKAcαR mice) were initially rederived from the Mutant Mouse Resource and Research Centers (MMRRC; stock no. 032825-MU, University of Missouri, Columbia, MO, USA) on a C57BL/6NCrl background and were maintained by breeding with C57BL/6J mice. Tg(*Six2*-EGFP/cre)1Amc mice (The Jackson Laboratory, Bar Harbor, ME, USA stock no. 009606) were maintained on a CD-1 background. Rosa26-CAG-LSL-tdTomato (Ai9) reporter mice were obtained from The Jackson Laboratory (stock no. 007909; 129S6/SvEvTac × C57BL/6NCrl background). *Six2*-Cre^+/−^ mice were crossed with Rosa26-CAG-LSL-tdTomato mice to generate *Six2*-Cre^+/−^;CAG-tdTomato^+/−^ offspring. These mice were subsequently crossed with PKAcαR^fl/wt^ mice. CA-PKA mutant mice with the following genotypes were used for the studies: *Six2*-Cre^+/−^;CAG-tdTomato^+/−^;PKAcαR^fl/wt^ and *Six2*-Cre^+/−^;CAG-tdTomato^−/−^;PKAcαR^fl/wt^. Littermate control mice with genotypes *Six2*-Cre^+/−^;CAG-tdTomato^+/−^ or *Six2*-Cre^+/−^;CAG-tdTomato^−/−^ were used as controls. For Figure 6A, four control and four mutant mice were analyzed. For the remaining experiments in Figure 6 and Figure 7, *n* = 3 (6–8 months of age) mice per group were used. Qualitative expression and localization studies (Figure 1, Figure 2, Figure 3, Figure 4 and Figure 5) were performed using two independent mice. A total of 18 mice were used. All animals were housed, maintained, and euthanized in accordance with the National Institutes of Health Guide for the Care and Use of Laboratory Animals. All experimental procedures were approved by the Tuskegee University Institutional Animal Care and Use Committee (Protocol# R01-2023-2). All mice were housed in temperature- and humidity-controlled rooms under standard laboratory conditions with a 12 h light/dark cycle. Environmental enrichment was provided in accordance with institutional guidelines.

### 4.2. Histology and Co-Immunofluorescence Microscopy

Co-immunofluorescence (co-IF) staining was performed as previously described in our published studies [39,64,65]. Stomachs were collected from control and mutant mice and fixed in 4% paraformaldehyde solution (Electron Microscopy Sciences, Morgantown, PA, USA, Cat # 15714) in PBS overnight at 4 °C. Fixed stomachs were processed in Sakura Tissue-Tek VIP series processor (Sakura Finetek USA, Torrance, CA, USA) and embedded in paraffin in the Immunohistopathology lab in TUCVM. Paraffin-embedded stomach tissues were sectioned at 4 µm thickness with Leica Microtome (Teaneck, NJ, USA). Sections were deparaffinized, rehydrated. For co-immunofluorescence staining, rehydrated sections were subjected to antigen retrieval in Tris-EDTA buffer (10 mM Tris Base, 1mM EDTA, 0.05% Tween-20, pH 9.0) at 95 °C for 30 min, followed by cooling at room temperature for 30 min (Millipore Sigma, St. Louis, MO, USA). Sections were blocked for 1 h at room temperature in donkey serum and incubated for 12–18 h at 4 °C with primary antibodies mentioned in Table 1 at the indicated dilutions. After washing 6 times with PBS, sections were incubated for 1 h at room temperature with the appropriate fluorophore-conjugated secondary antibodies described in Table 1. Images were acquired using a BioTek Lionheart FX Automated Microscope (Santa Clara, CA, USA).

### 4.3. Cellular Quantification and Statistical Analysis

Quantification of GDF9- and CHGA-positive cells in the control and CA-PKA stomachs was performed using the cellular analysis module of Gen5 Image Prime 3.11 software. For each section, a continuous tissue region of at least 500 µm in length was analyzed, and the corresponding tissue area (Object Sum Area) was determined based on the DAPI-positive region. The number of GDF9- and CHGA-positive cells was normalized to the measured tissue area. At least three independent control and three CA-PKA mutant mice were included. Quantification of CHGA-positive cells was analyzed using an unpaired two-tailed Student’s *t*-test using Prism 10.6.1 (799). Data are presented as mean ± SEM, and each mouse was considered an independent biological replicate.

## 5. Conclusions

In this study, we identified GDF9, a known paracrine regulator of ovarian folliculogenesis, NPHS1, a known regulator of insulin secretion, and RET, a neuronal marker involved in branching, as novel markers of the gastric neuroendocrine cell population. We also found that the gastric endocrine cell population is notably altered in the CA-PKA mouse model of gastric preneoplasia. Cellular quantification revealed a significant reduction in the density of GDF9- and CHGA-positive endocrine cells in CA-PKA stomachs, whereas NPHS1- and RET-expressing endocrine populations showed marked changes in their abundance and spatial distribution. These changes occur in parallel with extensive epithelial remodeling, suggesting that constitutively active PKA signaling in the gastric mesenchyme disrupts the gastric endocrine niche, likely through non-cell-autonomous mechanisms linked to mesenchymal–epithelial crosstalk. Moreover, the appearance of RET misexpression outside the endocrine compartment in CA-PKA mice suggests a potential contribution of aberrant RET signaling to pathological glandular remodeling and branching. Together, our findings expand the repertoire of molecular markers of gastric endocrine cells and uncover a previously unappreciated role for PKA-dependent mesenchymal–epithelial interactions in maintaining gastric endocrine homeostasis.

## Figures and Tables

**Table 1:** List of primary and secondary antibodies used.

Antibody	Company	Catalog #	Dilution
CHGA	Proteintech, Rosemont, IL, USA	23342-1-AP	1:2000
DDC	R & D Systems, Minneapolis, MN, USA	AF3564-SP	1:1000
ECAD	BD Biosciences, San Jose, CA, USA	610181	1:2000
GDF9	R & D Systems, Minneapolis, MN, USA	AF739	1:500
GHRL	R & D Systems, Minneapolis, MN, USA	MAB8200	1: 500
GIF	Millipore-Sigma, St-Louis, MO, USA	HPA040774	1:1000
MIST1	Cell Signaling, Danvers, MA, USA	14896	1:1000
Mouse IgG2a Isotype Control	R & D Systems, Minneapolis, MN, USA	MAB003	1:1000
Normal Goat IgG Control	R & D Systems, Minneapolis, MN, USA	AB-108-C	1:500
Normal Rabbit IgG Control	R & D Systems, Minneapolis, MN, USA	AB-105-C	1:1000
NPHS1	R & D Systems, Minneapolis, MN, USA	AF3159	1:500
Rat IgG1 Isotype Control	R & D Systems, Minneapolis, MN, USA	MAB005	1:500
Rat IgG2A Isotype Control	R & D Systems, Minneapolis, MN, USA	MAB006	1:500
RET	R & D Systems, Minneapolis, MN, USA	AF482	1:500
RFP	Rockland Immunochemicals, Pottstown, PA, USA	600-401-379	1:1000
RFP	Rockland Immunochemicals, Pottstown, PA, USA	200-101-379	1:500
SST	R & D Systems, Minneapolis, MN, USA	MAB2358	1:1000
Biotin Griffonia (Bandeiraea) Simplicifolia Lectin II (GSII)	Vector Labs Newark, CA, USA	BK-3000	1:1000
DyLight488 STREPTAVIDIN	Vector Labs Newark, CA, USA	SA-5488-1	1:1000
Alexa Fluor^®^ 488-conjugated AffiniPure Donkey Anti-Mouse IgG (H + L)	Jackson Immunoresearch, West Grove, PA, USA	715-545-150	1:1000
Donkey anti-Rabbit IgG (H + L) Highly Cross-Adsorbed Secondary Antibody, Alexa Fluor 488	Thermo Fisher Scientific, Milwaukee, WI, USA	A-21206	1:1000
Donkey anti-Rat IgG (H + L) Highly Cross-Adsorbed Secondary Antibody, AlexaFluor 488	Thermo Fisher Scientific, Milwaukee, WI, USA	A21208	1:1000
Alexa Fluor^®^ 594-conjugated AffiniPure Donkey Anti-Goat IgG (H + L)	Jackson Immunoresearch, West Grove, PA, USA	705-585-003	1:1000
Donkey anti-Rabbit IgG (H + L) HighlyCross-Adsorbed Secondary Antibody, AlexaFluor 594	Thermo Fisher Scientific, Milwaukee, WI, USA	A21207	1:1000
Donkey anti-Mouse IgG (H + L) HighlyCross-Adsorbed Secondary Antibody, AlexaFluor 594	Thermo Fisher Scientific, Milwaukee, WI, USA	A21203	1:1000

## Data Availability

The dataset is available in the NCBI Gene Expression Omnibus (GEO) under the accession number: GSE196236. The original contributions presented in this study are included in the article. Further inquiries can be directed to the corresponding author.
